# Fast first-photon ghost imaging

**DOI:** 10.1038/s41598-018-23363-w

**Published:** 2018-03-22

**Authors:** Xialin Liu, Jianhong Shi, Xiaoyan Wu, Guihua Zeng

**Affiliations:** State Key Laboratory of Advanced Optical Communication Systems and Networks and Center of Quantum Sensing and Information Processing (QSIP), Shanghai Jiao Tong University, Shanghai, 200240 China

## Abstract

Conventional imaging at low light levels requires hundreds of detected photons per pixel to suppress the Poisson noise for accurate reflectivity inference. We propose a high-efficiency photon-limited imaging technique, called fast first-photon ghost imaging, which recovers the image by conditional averaging of the reference patterns selected by the first-photon detection signal. Our technique merges the physics of low-flux measurements with the framework of computational ghost imaging. Experimental results demonstrate that it can reconstruct an image from less than 0.1 detected photon per pixel, which is three orders of magnitude less than conventional imaging techniques. A signal-to-noise ratio model of the system is established for noise analysis. With less data manipulation and shorter time requirements, our technique has potential applications in many fields, ranging from biological microscopy to remote sensing.

## Introduction

Photon-limited imaging has attracted great interest on account of its important applications under extreme environments, such as night vision^1^, biological imaging^2^, remote sensing^3^, and so forth, when off-the-shelf methods fail due to photon-limited data. Conventionally, the transverse spatial image is recovered by either a spatially resolving detector array with floodlight illumination or a single detector with raster-scanned point-by-point illumination. In this way, even with time-resolved single-photon detectors, hundreds of photons per pixel are necessary to suppress the Poisson noise that is inherent in photon counting to obtain accurate intensity values. At low light levels, an extremely long time and sufficient laser power are required for detection, which may result in failure of imaging. For example, in biological imaging^2,4^, samples may be destroyed by laser energy, or when the sample moves very quickly, the image cannot be well acquired^5–7^. At extremely low photon fluxes, the output from a single-photon detector consists of detections of a Poisson sequence of signal photons, background photons, and dark counts^8^. To suppress the Poisson noise, two methods have been mainly used: one is improving and perfecting the estimation model of the Poisson process^9–11^, and the other is designing a better measurement system^12,13^. For the former, many approaches based on wavelet-based methods^9,14,15^ were proposed. Later, more general and effective sparsity models have been investigated to deblur the Poisson noise, such as total variation, logarithmic regularization^14,16^, and the models based on image patches^15,17^. Kirmani *et al*.^18^ proposed a first-photon imaging(FPI) technique, which recovers the image from the first detected photon at each pixel. They reconstructed the scene reflectivity by maximizing the product of data likelihoods over all spatial locations combined with a sparsity-promoting regularization function^19^. Additionally, optimizing the design of the measurement system^13^ can facilitate the sensing capability. By exploiting the techniques of compressed sensing^12^ and ghost imaging (GI)^20–22^, Peter *et al*. obtained images from raw data comprised of fewer-than-one detected photon per pixel (PPP) by using an entanglement source^23^. With a classical source, Zeng’s group implemented computational imaging based on time-Correlated single-photon-counting (TCSPC-CI) at low light levels^24^. Edgar *et al*. realized 3D imaging with a single-pixel camera^25^ by histogramming the arrival times of the first backscattered photon of each illumination pulse for each illumination pattern, but that method still requires at least hundreds of detected PPP. Recently, we proposed first-photon ghost imaging (FPGI)^26^ approach, which estimates the reflection intensity by using the first detected photon of each illumination pattern, and can reconstruct an image using a PPP of no-more-than 1 on average. However, it still requires at least one photon detection in each measurement. The total detection time is limited by the frames with weakest light intensity, and the first photon of those frames need long time to detect.

Currently, conventional cameras or scanners for photon-limited imaging are limited by the resolution of the optical system or the scanning and detection times; on the other hand, the efficiency of ghost imaging is still limited by the measurement times and photon accumulation time for each measurement. In this article we present a high-efficiency photon-limited imaging technique, called fast first-photon ghost imaging (FFPGI), which exploits the conditional averaging scheme of correspondence imaging^27,28^ applied to the FPGI configuration. This method is much faster than conventional ghost imaging (CGI) because we only have to average over part of the reference data selected by the first-photon detection and do not have to compute the entire correlation functions. Furthermore, compared with first-photon imaging^18^, FFPGI has two significant advantages. First, there is no need to scan, an advantage gained from the spatial pre-modulation of the ghost imaging configuration. Secondly, there is no need to wait for the arrival of the first photon of each measurement because the information obtained is based on the pulse threshold rather than being dependent on the photon time-of-arrival records. That is undoubtedly a significant improvement compared to FPGI. The experiment results show that our scheme can reconstruct a 96 × 128 pixel image of around 8 dB PSNR(Peak signal-to-noise ratio) from 835 photon detection within 0.1 sec, which corresponding to 0.068 PPP. Our technique requires the least of photon detection per pixel compared with existing imaging techniques. The SNR model of the system is established for noise analysis, and the influence of the sparsity of reference patterns to the SNR is also discussed. With less data manipulation and shorter time required, our technique facilitates the practical applications of computational imaging that rely on sequential correlation measurements ranging from biological microscopy to remote sensing.

## Methods

### Imaging Set-Up

**Figure 1:**
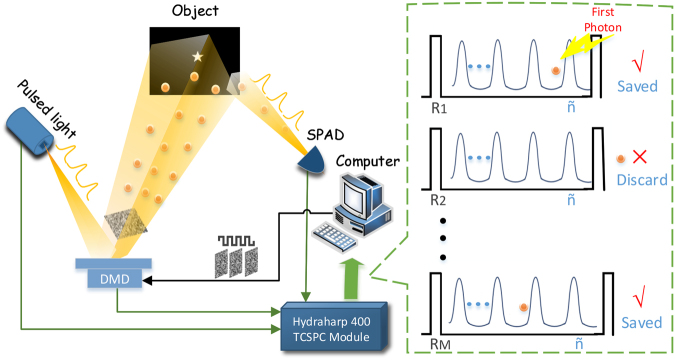
Schematic of FFPGI. The DMD modulates the spatial intensity of the pulsed light source with binary random speckle patterns. The reflected light from the object is detected by the SPAD. The dashed box shows the time sequence of the photon signals and synchronization signals recorded by HydraHarp 400. The computer is used to process data and to perform intensity correlation operations.

The imaging schematic is shown in Fig. 1. A super-continuum pulsed laser irradiates the programmable patterns of a digital micromirror device (DMD) and then illuminates the object. The DMD is an array of micromirrors consisting of 768 × 1024 independent addressable units for spatial modulation. At set intervals, the DMD controller loads each memory cell with the value ‘1’ or ‘0’, representing the illuminated or non-illuminated pixels at the object plane, respectively. In the experiment, the spatial modulation is conducted through a series of binary random speckle patterns, denoted by *R*_*i*_. Each pattern has 96 × 128 pixels, and each pixel consists of 8 × 8 micromirror units. The sparsity of these patterns, denoted by *Sp*, is the proportion of random ‘1’ among all pixels, and the sparsity of the target image is represented by *p*. For every illumination pattern, the first photon reflected from the object is recorded by a single photon avalanche diode (SPAD); the digital signal is then fed into the TCSPC module (HydraHarp 400), which receives synchronization signals from the DMD and pulsed light source. The number of pulses before the arrival of the first photon in the *i*_*th*_ sample pattern is recorded as *n*_*i*_. These first-photon data are used to estimate the intensity fluctuations of the different modulation patterns. Finally, the intensity correlation of the detected signals and the pre-modulated patterns are calculated by the computer.

### Imaging reconstruction

In low flux measurements, the individual photon detections obey Poisson statistics. Let *S* be the average number of laser photons arriving at the SPAD detector in response to single-pulse illumination, *B* be the arrival rate of background photons at the detector, *T*_*r*_ be the pulse repetition period, and *η* be the efficiency of the photon detection. Then the probability of no photons being detected in a single-pulse shot is

1 $${P}_{0}(S)={e}^{-\eta (S+B{T}_{r})},$$

where the background photons can be neglected when *T*_*r*_ is only 10^−6^ *s*. Because each pulse is independent, the number of pulses before the first detection, denoted by *n*, has a geometric distribution, i.e.,

2 $${P}_{{\rm{r}}}[{n}_{{\rm{i}}}=k]={P}_{0}{({S}_{{\rm{i}}})}^{k-1}[1-{P}_{0}({S}_{{\rm{i}}})].$$

The pointwise maximum-likelihood intensity estimate of Eq. 2, *Ŝ*_*ML*_, can be obtained by

3 $$\begin{array}{rcl}{\hat{S}}_{ML} & = & \mathop{{\rm{\arg }}\,{\rm{\max }}}\limits_{S\mathrm{ > 0}}\,\mathrm{log}\,\{{e}^{-\eta (k-\mathrm{1)}S}\mathrm{(1}-{e}^{\eta S})\}\\  & \approx  & \mathop{{\rm{\arg }}\,{\rm{\max }}}\limits_{S\mathrm{ > 0}}\,\mathrm{log}\,\{{e}^{-\eta (k-\mathrm{1)}S}(\eta S)\}\\  & = & \mathop{{\rm{\arg }}\,{\rm{\min }}\,}\limits_{S\mathrm{ > 0}}\eta \,(k-\mathrm{1)}S-\,\mathrm{log}\,\eta S.\end{array}$$

In Eq. 3, since *ηS* ≪ 1, the leading term in its Taylor series is used to approximate 1−*e*^−*ηS*^. The objective function defined in Eq. 3 is strictly convex. This computation yields

4 $${\hat{S}}_{ML}=\frac{1}{(n-\mathrm{1)}\eta }\propto \frac{1}{n}.$$

Assuming the reflection function of the object is *O*(*x*, *y*), the total intensity *S*_*i*_ with the *i*_*th*_ pattern illumination can be expressed as

5 $${{S}}_{{i}}\,=\,\iint {{R}}_{{i}}({x},\,{y}){O}({x},\,{y}){dxdy},$$

where *R*_*i*_(*x*, *y*) represents the modulation patterns, and (*x*, *y*) denotes the modulation region (i.e., the imaging region). According to the principle of the correlation measurements^20,29^, the second-order correlation function of the intensity fluctuation in signal arm with the spatial intensity distribution in reference arm reflects the image information. Thus, the object could be retrieved by the correlation measurements of the intensity estimation, 1/*n*_*i*_, with the pre-modulated patterns, *R*_*i*_(*x*, *y*),

6 $${{O}}_{{FPGI}}({x},\,{y})=\frac{1}{{M}}\sum _{{i}=1}^{{M}}\frac{\bar{{n}}}{{{n}}_{{i}}}({{R}}_{{i}}({x},\,{y})-\overline{{{R}}_{{i}}({x},\,{y})}),$$

where $\bar{n}$ is the average number of pulses before the first photon arrival, and M is the measurement times.

**Figure 2:**
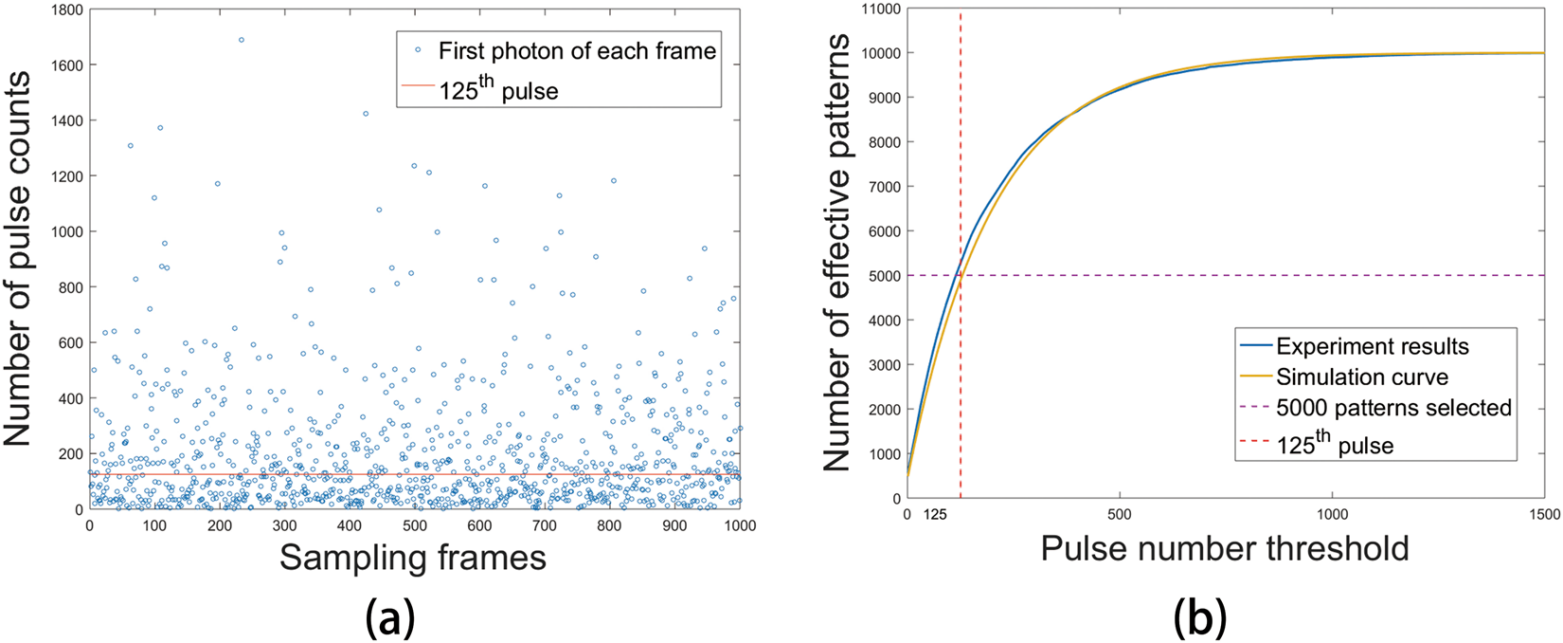
First-photon data collection and statistical processing. (**a**) Number of pulse counts of the first photons in 1000 measurements; red line is the 125th pulse. (**b**) Number of effective pattern frames versus pulse number threshold. Data below the red line in (**a**) and data on the left side of the red line in (**b**) correspond to the patterns selected for FFPGI.

Furthermore, the FFPGI is investigated by conditional averaging of random reference measurements. The threshold $\hat{n}$ is set to select the effective reference frames. If the first photon arrives before the $\hat{n}$th pulse of a certain pattern, this pattern will be identified as effective; otherwise, that pattern will be discarded. The proportion of patterns with the first-photon arrival before the pulse threshold satisfies Eq. (2). After the average photon counting rate has been measured, the corresponding theoretical curve can be plotted, as shown by the yellow line in Fig. 2(b). The proper pulse threshold can then be chosen according to the curve. Finally, the image is reconstructed by superposition of the effective patterns *R*_*j*_:

7 $${{O}}_{{FFPGI}}({x},\,{y})=\frac{1}{{K}}\sum _{{j}=1}^{{K}}({{R}}_{{j}}({x},\,{y})-\overline{{{R}}_{{j}}({x},\,{y})}).$$

Figure 2(a) shows the pulse counts of first photons in 1000 measurements. The photon counting rate in our experiment is 0.83%, i.e., there are 125 pulses on average before the arrival of the first photon. The experimentally measured and theoretical curves of the relationship between the number of frames that include a first-photon arrival and the pulse threshold are shown in Fig. 2(b); we see they agree well with each other. From the above plot, we can choose the 125th pulse as the threshold to select around 5000 relevant patterns in 10000 measurements. In practice, there is no need to detect any photons beyond the threshold, so FFPGI can save most time on the basis of FPGI.

We use the PPP to represent the photon efficiency for imaging, which can be expressed as

8 $${PPP}=\frac{{{N}}_{{ph}}}{{N}},$$

where *N*_*ph*_ is the number of photons actually measured and *N* is the total number of pixels in the retrieved picture. From the reconstruction method described above (see Eqs 6 and 7) we know that the PPP of FFPGI can be less than FPGI and FPI, because the value of *N*_*ph*_ in these three methods are: *K* < *M* < *N*. The lower limit depends on the least number of the superimposed patterns required by FFPGI. We note that the minimum value of PPP is restricted by the light transmission SNR, the detection efficiency, and the reconstruction algorithm.

### Noise Model

The spatially-structured light modulation is an essential step of our scheme. For certain illumination patterns, the number of pixels to be detected in a single measurement depends on the sparsity of the pattern, *Sp*. Since we use random binary patterns, the more the pixels illuminated the more the statistical noise in a single detection time. Let Δ*F* be the average amplitude fluctuation of a single pixel caused by the uncertain estimation of multi-pixels and *p* be the sparsity of the target image, so for retrieving an *N*-pixel object, the statistical fluctuation noise Δ*S*_*F*_ in each measurement can be expressed as:

9 $${\rm{\Delta }}{{S}}_{{F}}=\sum _{{x}}\sum _{{y}}{{I}}_{0}{\rm{\Delta }}{R}({x},\,{y}){O}({x},\,{y})={{I}}_{{0}}{({Sp}\times {N}\times {p}\times {\rm{\Delta }}{F})}^{{2}}.$$

Theoretically, decreasing the sparsity of the modulated patterns could increase the accuracy of intensity estimation. However, when the reference matrix sparsity is reduced to a certain level, the background noise instead of the random noise becomes the dominant factor. That is because the signal might be submerged by the white noise of the environment in a real scene during single-pixel detection. Let Δ*b*(*x*, *y*) be the white noise at each pixel and Δ*B* the average of Δ*b*(*x*, *y*). The background noise for a single pattern, denoted by Δ*S*_*B*_, can be described by,

10 $${\rm{\Delta }}{{S}}_{{B}}=\sum _{{x}}\sum _{{y}}{\rm{\Delta }}{b}({x},\,{y})={N}\times {\rm{\Delta }}{B}.$$

Therefore, for each measurement, the SNR of the imaging system is given by

11 $$\begin{array}{rcl}SNR & = & 10\,{\rm{lg}}\,\frac{\sum _{x}\sum _{y}{I}_{0}R(x,y)O(x,y)}{\sum _{x}\sum _{y}{\rm{\Delta }}{I}_{0}R(x,y)O(x,y)+\sum _{x}\sum _{y}{I}_{0}{\rm{\Delta }}R(x,y)O(x,y)+\sum _{x}\sum _{y}{\rm{\Delta }}b(x,y)}\\  & = & 10\,{\rm{lg}}\,\frac{{I}_{0}Sp\cdot p\cdot N}{{\rm{\Delta }}{I}_{0}Sp\cdot p\cdot N+{\rm{\Delta }}{S}_{F}+{\rm{\Delta }}{S}_{B}}\\  & = & 10\,{\rm{lg}}\,\frac{{I}_{0}}{{\rm{\Delta }}{I}_{0}+{I}_{0}\cdot Sp\cdot N\cdot p\cdot {\rm{\Delta }}{F}^{2}+\frac{{\rm{\Delta }}B}{p\cdot Sp}}\end{array},$$

where *p* is the percentage of high-reflectivity pixels of the object. From Eq. 11, we know that the trade-off between the statistical noise and background noise gives rise to a peak in the curve, because in the denominator the second term increases with increasing sparsity but the third term decreases.

### Roadfilter algorithm

To avoid the background noise photons being detected we can set a time gate for the photons’ time of flight (TOF) to filter out the noise photons that are not reflected from the object plane. This is because the TOF represents the different distances of the reflected light source planes to the detection plane. Moreover, the Roadfilter algorithm^18^ can be used to deblur the background noise and enhance the visibility of the image after the original reflectivity estimation is finished. This method exploits the natural spatial correlation of the objects. First, the rank-ordered absolute difference statistics of a certain spatial point is computed by using the intensity of its eight neighboring pixels, then a binary hypothesis test is conducted by using a threshold to identify whether the photon detection was due to signal or noise. This threshold is dependent on the original intensity reconstruction by Eqs 5 and 7.

**Figure 3:**
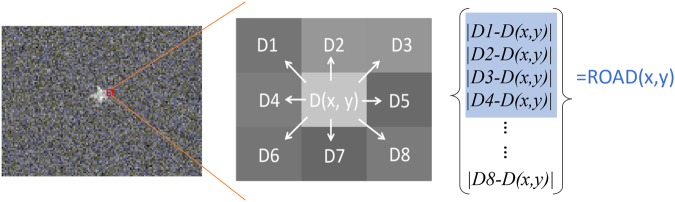
The schematic of ROAD filter algorithm.

As shown in Fig. 3, at each transverse location (*x*, *y*), the ROAD statistic is first computed using the reflectivity estimation of the eight nearest transverse neighbors, denoted by *D*_1_–*D*_8_. The absolute intensity differences: $|{D}_{1}-D(x,y)|$, …, $|{D}_{8}-D(x,y)|$ are sorted in ascending order, and the ROAD statistic ROAD (*x*, *y*) is the sum of the first four absolute differences from this sorted collection. The pixels of the reconstructed image have been normalized. The fluctuation of the intensity estimation Δ*D* for each pixel is 0.1, which depends on the experimental conditions. Let *Round* denote the operator for the nearest integer. Then, the intensity of the point *D*(*x*, *y*) is updated as follows:

$$\begin{array}{c}{\rm{if}}\,{\rm{ROAD}}\,(x,\,y)\ge 4\Delta D,\,D(x,y)=Round{D}_{0}(x,y)\\ {\rm{if}}\,{\rm{ROAD}}\,(x,\,y)\le 4\Delta D,\,D(x,y)=1-Round{D}_{0}(x,y)\end{array}$$

## Results

**Figure 4:**
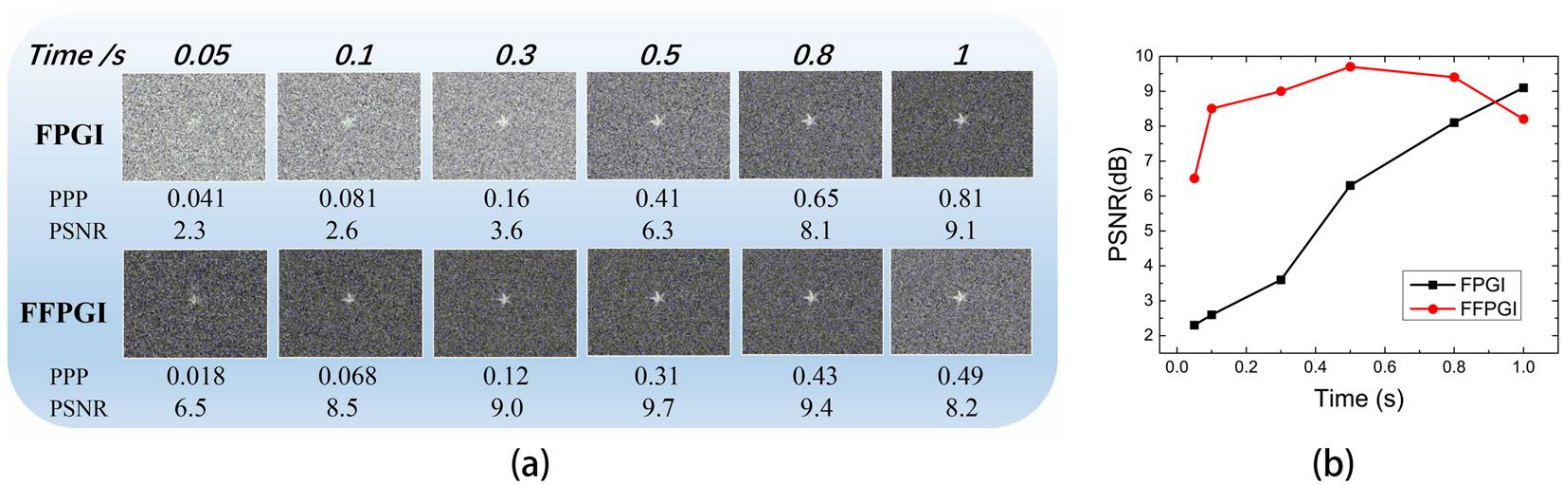
Experimental results showing the dependence of FPGI and FFPGI on integration time. (**a**) FPGI results for measurement times of 500, 1000, 3000, 5000, 8000, 10000; FFPGI results with the variable pulse-count thresholds: 5, 10, 30, 50, 80, 100 for 10000 measurements. (**b**) PSNR curves versus the integration time.

The experimental results of the dependence of FPGI and FFPGI upon the integration time are shown in Fig. 4(a). With the increase of the PPP, the details of the object become more distinct for both FPGI and FFPGI, while the background noise in the latter becomes more evident. This is because more patterns are overlapped as the number of first-photon detections increases in FFPGI, and more background noise enters. From Fig. 4(b), FFPGI only requires 0.1 sec to retrieve an 8 dB PSNR image, which is 7/8 times shorter than the time cost of FPGI. With a suitable pulse number threshold, a clear image can be obtained by this extremely concise method. The experimental results demonstrate that FFPGI can retrieve a 96 × 128 pixels image from less than 1000 first-photon data, which corresponds to <0.1 PPP.

**Figure 5:**
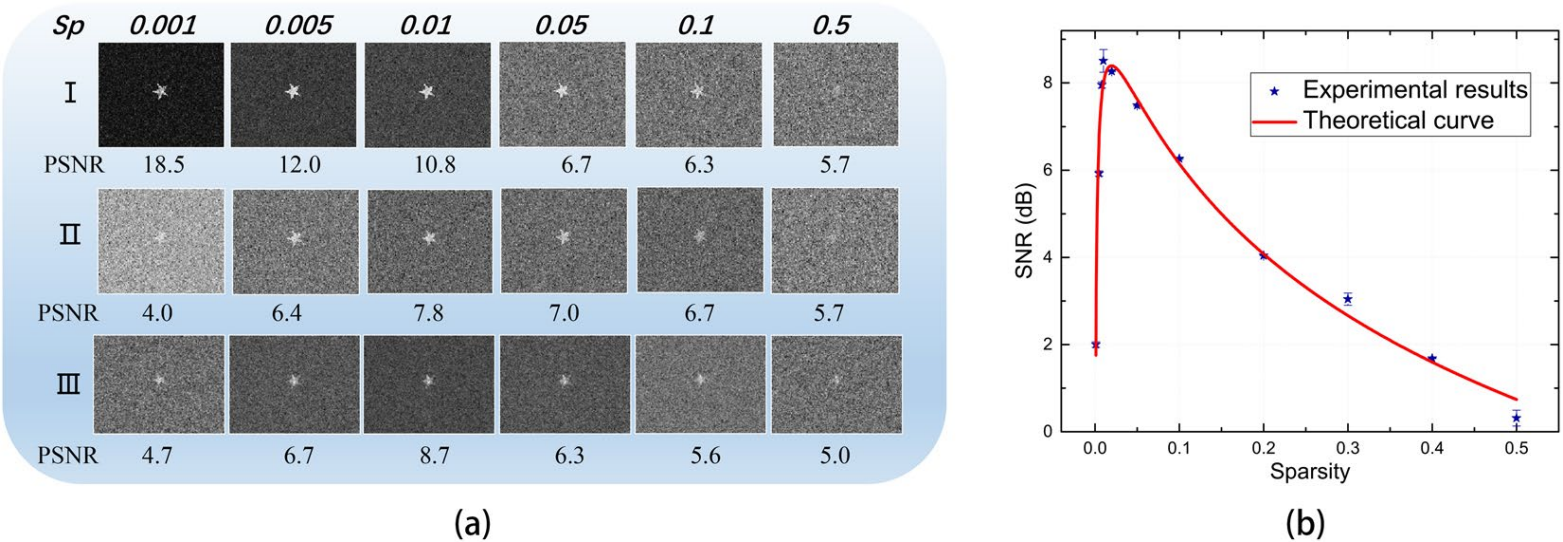
Simulation and experimental reconstructions from 10000 measurements for sparsity values of the reference pattern from 0.001 to 0.5. (**a**) I, II: Simulation reconstructions without (I)/with (II) background noise. III: Experimental reconstruction with background noise; (**b**) Theoretical SNR curve (red line) from Eq. 11 and experimental SNR results (blue stars). The parameter values of *I*_0_, *p*, Δ*I*_0_, Δ*F* and Δ*B* in Eq. 11 are 10, 10^−2^, 5 × 10^−2^, 10^−1^ and 5 × 10^−5^, respectively.

The sparsity of the modulation matrix is adjusted from 0.001 to 0.5, and the corresponding results of both simulation and experiment by FFPGI are shown in Fig. 5. The simulation results without background noise show that the restored image quality becomes higher as the modulation sparsity decreases, while both experiment and simulation show that with background noise too low a sparsity also reduces the reconstruction quality.

The theoretical and experimental SNR values versus the sparsity of the modulated patterns are shown in Fig. 5(b), and they agree well with each other. The optimal point, 0.01 sparsity, results from the trade-off between the statistical noise during modulation and the background white noise. These experimental results substantiate the noise model of Eq. 11.

**Figure 6:**
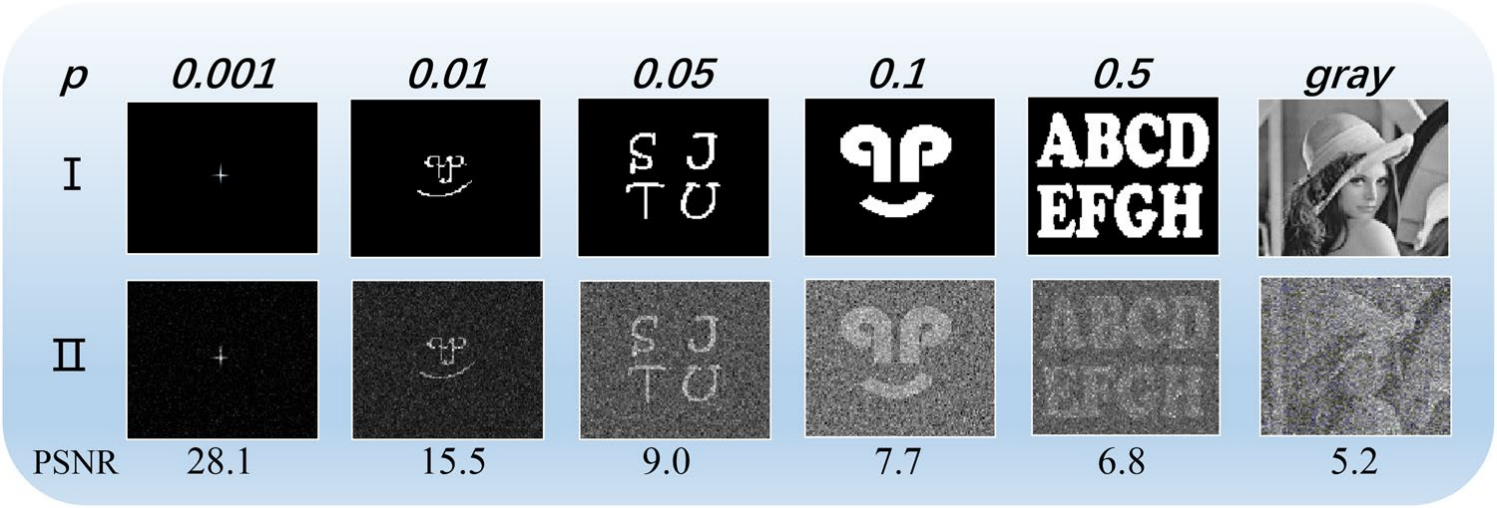
Image reconstruction results obtained with 10000 measurements for a target sparsity range from 0.001 to 0.5; the last object is a gray image. All images were reconstructed with their optimal sparsity values.

We can see that the target images have different properties in Fig. 6. The restored images become blurred as the sparsity increases. On the other hand, FFPGI can retrieve the outline of the target even in the greyscale picture. The performance can be improved by some further variations of the reconstruction algorithm, such as through the use of the standard numerical solver of reference^19^.

**Figure 7:**
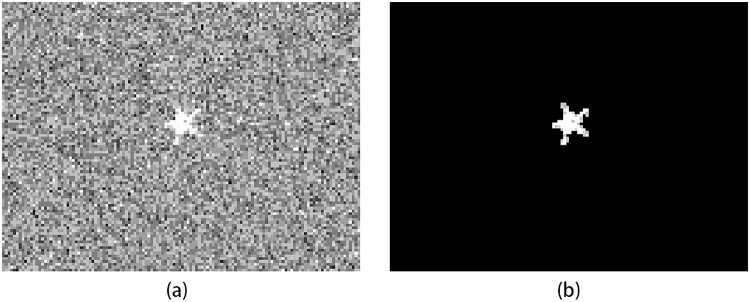
(**a**) The original reconstruction by FFPGI; (**b**) the deblurred result obtained by using the Roadfilter algorithm. The reconstruction exploited 3000 first-photon data in 10000 measurements, the pulse number threshold being the 50th pulse.

Figure 7 shows the original reconstruction by FFPGI and the result after the Rodfialter post-treatment. By iterating the Roadfilter algorithm several times, almost all background noise can be eliminated.

Table 1 gives a comparison of CGI, FPI, FPGI and FFPGI by reconstructing a 96 × 128 pixel image. The PPP of FPI is obtained from ref.^18^, and other three are from our experimental data. The detection time is calculated assuming a photon counting rate of 0.83% and a laser pulse repetition rate of 1 MHz (i.e., there are 125 pulses on average before the arrival of the first photon). From Table 1, FPGI and especially FFPGI have a higher photon efficiency than the previous two techniques, judging by the PPP. Furthermore, FFPGI only requires a fixed single-pixel detector without any spatial resolution and a fixed pulsed light source.

**Table 1:** Comparison of CGI, FPI, FPGI and FFPGI.

Condition	CGI	FPI	FPGI	FFPGI
PPP	10^2^–10^3^	≥1	0.65	0.068
Detection time (s)	20	1.5	1	0.1
Detector	single-pixel	scanner	SPAD	SPAD

## Discussion

The FFPGI technique can achieve high-efficiency performance at the extremely low light levels with only a fixed single-pixel detector. It requires merely one-eighth of the time required by FPGI, and is much faster than conventional computational imaging. Additionally, we have proposed an SNR model is established to analyze the noise components that affect the image quality. Our technique can extract more spatial and temporal information from the collection of single detection data compared with existing imaging methods. Thus, a lot of time as well as laser power can be saved, which facilitates multi-scenario imaging under photon-limited conditions. It is even superior for remote sensing in the ocean and aerial surveys as well as for biomarker recognition in dark fields under the microscope, when recognizing a small object in a wide field of view requires high efficiency and accuracy. This technique can be applied to enhancing the performance of computational imagers that rely on sequential correlation measurements.

Our FFPGI system can be further improved by incorporating existing techniques based on the framework of computational GI, including multicolor GI^30,31^ and target tracing^32,33^. These techniques realize image restoration under various scenarios by using multiplexing modulation and detection, adaptive speed retrieval and various excellent recovery algorithms. Our approach can be combined with these imaging schemes as a technique for efficiency enhancement, and thus achieve multi-scenario imaging under photon-limited conditions. By utilizing the arrival-time data of the first photon for range dimension sensing or multi-point detection^34^, this scheme can also be used for 3D imaging. Moreover, the photon efficiency of FFPGI can be further improved by exploiting compressed sensing algorithms. Further research will be conducted in the future.
